# Exploring non-surgical alternatives for low to intermediate-grade *in situ* ductal carcinoma of the breast using vacuum-assisted excision: the VACIS protocol

**DOI:** 10.3389/fmed.2024.1467738

**Published:** 2024-09-24

**Authors:** Luca Nicosia, Luciano Mariano, Antuono Latronico, Anna Carla Bozzini, Federica Bellerba, Aurora Gaeta, Filippo Pesapane, Giovanni Mazzarol, Nicola Fusco, Giovanni Corso, Claudia Sangalli, Cristian Gialain, Matteo Lazzeroni, Sara Raimondi, Enrico Cassano

**Affiliations:** ^1^Department of Biotechnology and Life Sciences, University of Insubria, Varese, Italy; ^2^Breast Imaging Division, European Institute of Oncology (IEO), Istituto di Ricovero e Cura a Carattere Scientifico (IRCCS), Milan, Italy; ^3^Molecular and Pharmaco-Epidemiology Unit, Department of Experimental Oncology, European Institute of Oncology (IEO), Istituto di Ricovero e Cura a Carattere Scientifico (IRCCS), Milan, Italy; ^4^Department of Statistics and Quantitative Methods University of Milano-Bicocca, Milan, Italy; ^5^Division of Pathology, European Institute of Oncology (IEO), Istituto di Ricovero e Cura a Carattere Scientifico (IRCCS), Milan, Italy; ^6^Department of Oncology and Hemato-Oncology, University of Milan, Milan, Italy; ^7^Division of Breast Surgery, European Institute of Oncology (IEO), Istituto di Ricovero e Cura a Carattere Scientifico (IRCCS), Milan, Italy; ^8^Clinical Trial Office, European Institute of Oncology (IEO), Istituto di Ricovero e Cura a Carattere Scientifico (IRCCS), Milan, Italy; ^9^Division of Cancer Prevention and Genetics, European Institute of Oncology (IEO), Istituto di Ricovero e Cura a Carattere Scientifico (IRCCS), Milan, Italy

**Keywords:** ductal carcinoma *in situ*, vacuum-assisted biopsy, breast neoplasms, biopsy, stereotactic, minimally invasive surgical procedures

## Abstract

**Background:**

Surgery is still the standard treatment for breast lesions such as *in situ* ductal carcinoma (DCIS); however, its survival benefit is minimal, particularly for low-grade DCIS. Surgical complications and related depression status can adversely affect patients’ quality of life. Approximately 25% of breast cancer (BC) cases are *in situ* forms, with DCIS making up 90% of these. Low and intermediate-grade DCIS often grow slowly and do not always progress clinically significant diseases. Identifying non-invasive lesions could help prevent overtreatment. In this context, new diagnostic tools like vacuum-assisted excision (VAE) could enhance the management of these conditions.

**Methods:**

The prospective VACIS study explores the role of VAE in ensuring the absence of pathology at subsequent surgery and reducing the diagnostic underestimation of breast biopsies for microcalcifications. Patients with suspicious breast microcalcifications up to 15 mm, who are candidates for stereotactic biopsy, will be enrolled and randomised into two groups. The control group will complete the biopsy with typical sampling, aiming to collect some microcalcifications from the target, while the experimental group will focus on the complete removal of the biopsy target (confirmed by mammography on the biopsy table), followed by a second sequence of cleaning samples. Radiograms will confirm lesion removal. Pathologic outcomes at surgery will be compared between the groups, and the percentage of underestimation will be assessed. The sample size is calculated to be 70 patients per group, using statistical tests and multivariate logistic models to detect a significant difference in the absence of pathology. Data collected will include patient age, lesion characteristics, and details of the biopsy, pathology and surgery.

**Discussion:**

Current surgical treatments for low-and sometimes intermediate-grade DCIS offer limited survival benefits and may hurt patients’ quality of life due to surgery-related complications and associated depression. These lesions often grow slowly and might not become clinically significant, suggesting a need to avoid overtreatment. Improved diagnostics procedures, such as VAE, could help distinguish non-invasive from potentially invasive lesions, reduce biopsy underestimation, enable personalised management and optimise treatment strategies. This study hypothesises that VAE could be a viable alternative to surgery, capable of removing pathology during the biopsy procedure.

**Clinical trial registration:**

Clinicaltrials.gov, identifier NCT05932758.

## Introduction

The advent of breast mammography screening has significantly increased the diagnosis of *in situ* breast lesions (1). Approximately 25% of all BC cases are *in situ*, meaning the cancerous growth is confined to the duct or lobule and is generally clinically occult with minimal potential for spread. Notably, DCIS accounts for 90% of *in situ* BCs (2). Managing these lesions represents a significant economic and health challenge.

Surgery remains the standard for breast lesions like DCIS (3). Over 98% of patients diagnosed with DCIS undergo surgical procedures, often in combination with radiotherapy (RT). However, most cases rarely progress to invasive BC, with a mortality rate of only 4% (4). The risk of progression and aggressiveness is associated with disease grade, with high-grade tumours linked to a poorer prognosis (4). Research suggests that greater aggressiveness is also related to multifocality and abnormal branching and lobularization, known as neoductgenesis (4). Identifying these patterns through imaging and histology may help recognise inherent aggressiveness and tailor treatment accordingly. Thus, aggressive treatment for DCIS, especially in patients with additional health issues, might constitute overtreatment (5). The survival benefit of surgical resection in these patients is low, especially for low-grade DCIS, where surgical comorbidities and prior depression are linked to a poorer quality of life (6). Low-grade and sometimes intermediate-grade DCIS typically exhibit slow growth and often do not progress to clinically significant disease (6). Therefore, distinguishing harmless lesions from potentially invasive ones can help avoid overtreatment in many patients. Nonetheless, the public health costs of surgery and prolonged follow-up remain comparable.

Recent studies have focused on identifying less aggressive forms of *in situ* lesions to enable the de-escalation of conventional treatments (4). A growing interest is in personalised medicine tailored to the patient’s specific pathology. Four international prospective study protocols (LORIS, COMET, LORD, and LORETTA) are currently evaluating non-invasive treatment methods for DCIS (7), primarily aiming to assess the effectiveness and safety of active surveillance compared to surgery-based approaches for low-risk DCIS patients.

Our study protocol (VACIS protocol) aims to identify a population of patients for whom non-surgical management can be proposed more safely. Enhancing the effectiveness of active surveillance involves reducing the upgrade rate where DCIS confirmed by presurgical biopsy is later reclassified as invasive carcinoma in surgical specimens (8). Data on the underestimation rate of DCIS diagnoses is contentious. A significant meta-analysis by Brennan et al. (8) found that up to 26% of patients with biopsy-confirmed DCIS had synchronous invasive carcinoma in surgical specimens. Consequently, efforts should focus on identifying factors that lead to biopsy underestimation (infiltrating tumours diagnosed as DCIS) and identifying patients at risk of developing the invasive disease during follow-up. Current evidence suggests that new diagnostic and interventional techniques, such as VAE, can significantly reduce the rate of diagnostic underestimation and help identify patients with distinct disease progression, allowing for personalised management.

VAE involves the removal of at least 4 grams of breast tissue using a vacuum-assisted biopsy (VABB). In our study protocol, we have standardized the procedures by dividing them into an initial sequence of extractions that remove the lesion and a second sequence of extractions, referred to as “cleaning,” akin to a surgical procedure. The primary aim of this study protocol is to investigate the relationship between the role of VAE and the lack of pathology *in situ* at subsequent surgery. Could VAE substitute surgery in some patients with low-grade DCIS categories? Since most *in situ* forms are microcalcifications, the study protocol is focused exclusively on stereotactic biopsies. The secondary aim is to evaluate the role of VAE in reducing the diagnostic underestimation of the breast biopsy with a histological result of DCIS.

### Ethics and consent

The study protocol has been evaluated and authorised by the European Institute of Oncology ethical (number of approval IEO 1856) committee. Participants will sign written informed consent, and researchers will adhere to institutional data collection and management guidelines.

## Methods and analysis

The study is a two-arm trial involving 300 patients (*n* = 150 in the VAE group and *n* = 150 in the non-VAE group) over 36 months. Participants are randomly assigned to either the VAE (intervention) or standard-of-care (control) group.

In the control group, biopsies will follow the typical sampling procedure aimed only at collecting some microcalcifications from the target. In the VAE group, the focus will be on the complete removal of the biopsy target, confirmed by mammography on the biopsy table, followed by a second sequence of cleaning samples. Radiograms will confirm lesion removal for the intervention group. Pathology analysis will report separately each sample. Pathological outcomes at surgery will be compared between the two groups. To adhere to the protocol, it is necessary to have a digital stereotactic biopsy guidance system (with a dedicated patient table).

A statistical comparison will be performed to evaluate the differences between the VAE and Standard-of-care groups.

### Study design

All patients with suspicious breast lesions (BI-RADS ≥3) who are candidates for stereotactic VABB and whose lesions present as microcalcifications (the most common form of low and intermediate-grade DCIS) will be prospectively selected. The radiologists involved in the procedure are all dedicated to breast imaging and have at least 5 years of work experience. Patients with a mammographic lesion measuring less than 15 mm in diameter will be enrolled and randomly assigned into two groups on a 1:1 basis. The randomization data will be securely stored on a password-protected computer at the European Institute of Oncology, accessible only to researchers involved in the study.

In the control group, biopsies will focus solely on sampling a few microcalcifications (fewer than 12 samples) and may not necessarily use an 8G needle. In the VAE group, the lesion will be macroscopically removed using an initial sequence of at least eight samples with an 8G needle, followed by a cleaning sequence of at least four samples. This group will undergo an excisional biopsy (VAE) according to United Kingdom guidelines, which define VAE as a collection of at least 4 grams of tissue with an 8G needle. The VAE will be performed during the same biopsy session without recalling the patient later. Both groups will receive post-procedure mammograms and specimen radiograms. The specimen radiogram is used to separate the specimens with microcalcifications from those without, to assist the pathologist, and to verify that the biopsy procedure has indeed sampled the target microcalcifications. We also have the ability to obtain mammographic images while the patient is still on the biopsy table. For the interventional group, if residual microcalcifications are present, the sampling will continue until they are completely removed. For the control group, the goal is not the complete removal of the target but rather the correct diagnostic procedure, so even if residual microcalcifications remain after the biopsy, the procedure will be concluded.

All patients with low and intermediate-grade DCIS histological findings will be included in the study and are expected to be evenly distributed between the VAE and control groups. These patients will undergo surgery, and we will compare the percentage of patients with no pathology (including *in situ*) at the surgery between the two groups to determine if VAE can effectively remove small lesions and potentially serve as an alternative to surgery. The surgical specimens will be radiographed to verify the presence of the post-biopsy clip in the surgical sample. We will also compare the rate of biopsy underestimation of invasive carcinoma between the groups. The surgical tissue sections will be documented to ensure consistency between the two groups.

**Figure 1:**
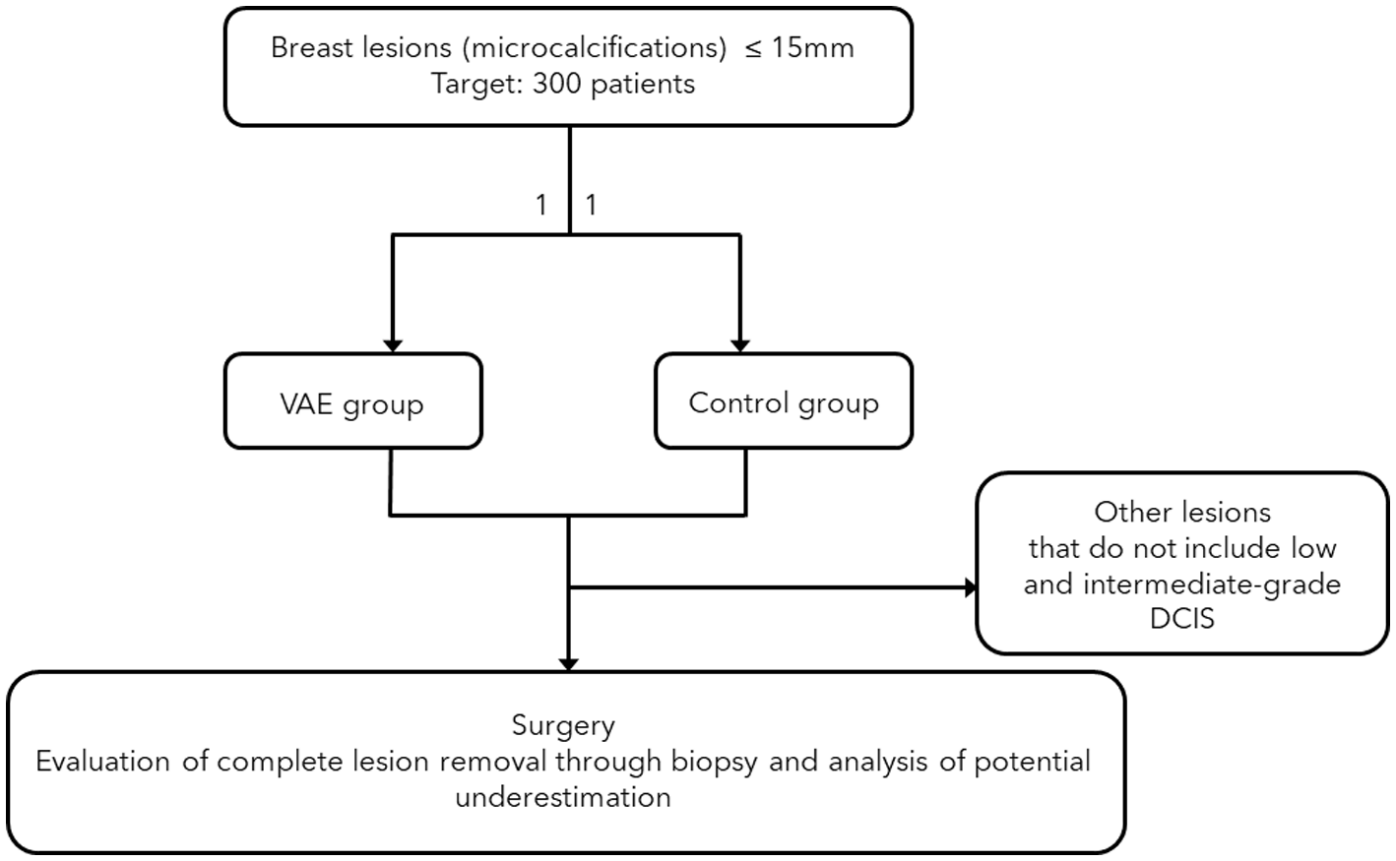
Study design.

Our goal is to enroll at least 300 patients in this study. We focus on stereotactic VABB because *in situ* neoplasms predominantly present as microcalcifications, and complete macroscopic removal of the lesion can be more easily verified with mammography rather than ultrasound. The study design is summarized in Figure 1.

#### Inclusion criteria

1. Patients with suspicious breast lesions (BI-RADS ≥3) presenting as microcalcifications.
2. Patients capable of and willing to comply with the specific informed consent form.
3. Patients undergoing stereotactic VABB.
4. Patients with low to intermediate-grade DCIS following biopsy.
5. Patients scheduled for surgery.

#### Exclusion criteria

1. Patients undergoing other types of biopsies (not stereotactic).
2. Patients whose lesions are not in the form of microcalcifications.
3. Patients who refuse to sign the specific informed consent.
4. Patients whose histological biopsy result is not low/intermediate-grade DCIS.
5. Patients with multifocal and/or multicentric lesions.
6. Patients who will not undergo surgery.
7. For the VAE group only: patients in whom haematoma prevents the continuation of the second sequence of biopsies.

The data to be recorded during the biopsy procedure are listed in Table 1.

**Table 1:** Data recorded during the biopsy procedure.

Characteristics	
**Age (years)**	**Needle (Gouge)**
**Menopausal status**	7G
Pre-	8G
Post-	10G
**Lesion side**	11G
Right	**Samples (n)**
Left	**Lesion excision**
**Lesion site**	Minimal
Upper-outer	Partial
Lower-outer	Complete
Upper-inner	**Clip**
Lower-inner	Yes
Retroareolar	No
Inner-equatorial	**Clip placement**
Outer-equatorial	On-site
Upper-hemitelic	Dislocated
Lower-hemitelic	**Clip dislocation (from the sampling site)**
**Lesion size (mm)**	Distance (mm)
**Lesion type**	Sector (upper, lower, outer, inner)
Microcalcifications	**VAE**
Architectural distortion	Yes
Lump	No
Asymmetry	**Complications**
**VABB type**	Yes (additional notes)
Stereotactic	No
Tomographic	**Histological findings**
Ultrasound	

### Statistical analysis

The primary endpoint is to compare the percentage of patients with no detectable pathology at surgery between the group undergoing standard treatment (macroscopical removal of the lesion) and the group undergoing VAE (VAE group). Based on the assumption that macroscopical removal without VAE will result in approximately 20% of patients having no pathology at the subsequent surgery, a sample size of 70 patients per arm is calculated to achieve 90% power to detect a 16% difference between the two treatment groups.

This calculation is based on an inequality test for two independent proportions, assuming a 0.2 proportion under the null hypothesis (standard treatment group) and a 0.04 proportion under the alternative hypothesis (VAE group). The test statistic will be the two-sided Z test with pooled variance, using a significance level 0.05.

Continuous variables will be summarized with the median and interquartile range for each treatment arm, while categorical variables will be presented as frequency and percentage. Differences between the two groups will be evaluated using the Wilcoxon rank-sum test for continuous variables and the chi-square test for categorical variables. Multivariate logistic regression models will be used to assess the association between the treatment group and the absence of pathology at surgery, adjusting for key clinical variables and potential confounders. The results will be quantified in terms of odds ratios (Ors), with statistical significance evaluated using *p*-values and a significance threshold set of 0.05.

## Discussion

DCIS is currently treated aggressively due to the challenge of predicting which cases will progress to invasive BC (9). Standard treatment typically involves surgical excision, and patients opting for breast-conserving surgery often receive adjuvant local RT to reduce the risk of local recurrence (10). Additionally, adjuvant endocrine therapy May be recommended based on the tumour’s hormone-receptor status to minimize further the recurrence risk in patients undergoing breast conservation (11). Long-term survival rates for DCIS remain high regardless of the surgical approach or use of RT, with cause-specific survival rates of 98.9% at 10 years and 96.7% at 20 years (12).

There is growing interest in reducing the intensity of DCIS treatment due to several conditions. DCIS has a low BC-specific mortality rate, whether treated with standard methods or potentially even without treatment (13). A SEER study of 108.196 DCIS patients treated with standard methods reported BC-specific mortality rates of 1.1% at 10 years and 3.3% at 20 years (14). Autopsy studies have found undiagnosed DCIS in up to 39% of women who died from non-BC causes, indicating that DCIS can remain non-aggressive and May not always progress to significant disease in some women (15). Moreover, the widespread use of mammography has led to increased detection of DCIS, but this has not corresponded to a decreased incidence of invasive cancer, suggesting that DCIS may not always evolve into invasive carcinoma (16).

A major focus in reducing DCIS treatment is identifying women who might not require surgery after diagnosis. These patients could be managed with active surveillance, potentially with or without endocrine therapy. Four phase III prospective trials are exploring active image-based surveillance for patients with low-risk DCIS: COMET in the United States, LORD in Europe, LORETTA in Japan, and LORIS in the United Kingdom (17). The trials primarily aim to monitor the progression to ipsilateral invasive cancer. Due to low enrollment, the LORIS and LORD trials have transitioned to registry studies (18).

We propose that radiology can play a crucial role in reducing the diagnostic underestimation of biopsies and, through this study protocol, position itself as a viable alternative to surgery.

This approach can help identify patients for whom active surveillance is a safe option.

Our study aims to identify patients for whom active surveillance can be safely recommended, thereby increasing the enrollment in non-operative management of DCIS. The role of VAE as a potential alternative to surgery has already been successfully investigated for benign lesions and, in preliminary stages, for high-risk lesions (19). We believe that its role in managing *in situ* lesions could be equally promising.

One of the study’s strengths is the integration of VAE on the same day and during the same session as the conventional biopsy procedure. This method offers significant time and economic savings and reduces psychological stress for the patient. However, this protocol will likely result in performing VAE in many patients who do not have *in situ* pathology, as the histological outcome is not known at the time of the biopsy. We believe the risks associated with VAE are minimal and not significantly higher than those of the standard stereotactic procedure. Anyway, the percentage of hematomas and peri-biopsy complications will be recorded and compared between the two groups.

## Conclusions ethics and dissemination

The study protocol proposed in our project is simple, easily implementable, and exposes patients to minimal risk. Should the ability of VAE to remove the *in situ* pathology in a high percentage of cases and to negligibly reduce diagnostic underestimation be confirmed, extremely important benefits would be obtained for public health and for patients with *in situ* breast carcinoma, aiming for increasingly effective and personalized therapeutic management.

From an ethical standpoint, the protocol is easily implementable and exposes patients to minimal risk. From a dissemination perspective, the protocol is simple for those involved in breast interventional procedures and can be easily reproduced in many centers.
